# Prognostic Value of Cancer-Associated Fibroblast-Related Gene Signatures in Hepatocellular Carcinoma

**DOI:** 10.3389/fendo.2022.884777

**Published:** 2022-06-06

**Authors:** Wenge Dong, Yangyang Xie, Hai Huang

**Affiliations:** Department of General Surgery, Hangzhou Traditional Chinese Medicine (TCM) Hospital Affiliated to Zhejiang Chinese Medical University, Hangzhou, China

**Keywords:** cancer-associated fibroblasts (CAFs), hepatocellular carcinoma (HCC), chemosensitivity, immunotherapy response, tumor microenvironment

## Abstract

Hepatocellular carcinoma (HCC) is a global health challenge with an increasing incidence worldwide. Cancer-associated fibroblasts (CAFs) function critically in HCC initiation and development. However, the prognostic significance of CAF-related gene signatures in HCC remains unknown. Therefore, the specific functions of CAF-related genes in HCC were investigated to help develop potential therapeutic strategies. In this study, CAF-related genes were screened from three CAF-related gene sets. HCC data from the Gene Expression Omnibus (GEO) database was applied to verify the screened CAF-related genes. Cluster analysis was used to identify clusters based on the expression pattern of CAF-related genes and two identified clusters were found to have a significant difference in overall survival (OS) and progression free intervals (PFI). The prognosis of HCC patients was predicted using the prognostic risk score model developed based on HCC data from The Cancer Genome Atlas (TCGA) databases. High-risk group patients had a worse OS than those in low-risk group in TCGA. These results were validated in International Cancer Genome Consortium (ICGC) database. Moreover, combining the clinicopathological characteristics related to prognosis with the model, a nomogram was built for a more accurate prediction of OS of HCC patients. In addition, analyses of immune infiltration characteristics of tumor microenvironment (TME), chemosensitivity, and immunotherapy response were conducted to further evaluate the prognostic value of CAF-related genes. Patients with low-risk scores were found to have higher chemosensitivity to cisplatin, doxorubicin, and sorafenib. Individuals with high-risk scores were found with a higher expression of most immune checkpoints which indicated patients with high-risk scores may benefit more from treatment with immune checkpoint inhibitors. Furthermore, a correlation between immune infiltration characteristics of TME and patients with different risk levels was found. These findings provide a possibility for the further development of personalized treatments in HCC.

## Introduction

Liver cancer has experienced an increasing incidence worldwide in recent decades (1, 2). By 2030, more than 1 million people will die from liver cancer according to World Health Organization estimate (3). As the most prevalent type of liver cancer, hepatocellular carcinoma (HCC) accounts for about 90% of all liver cancer cases. Despite new treatment methods for HCC, the cancer prognosis remains poor (4). Therefore, there is an urgent need to develop a new promising target for therapies and an effective prognostic model for patients suffering from HCC.

The malignant nature of cancer growth is controlled by paracrine communication between the tumor and surrounding stroma (5). Fibroblasts, the primary cell type inside the stroma, are known as cancer-associated fibroblasts (CAFs) and orchestrate interaction with cancer cells (6, 7) as well as exhibit various prognostic markers (8). Increasing research suggests that CAFs are essential to HCC development (9–12). Furthermore, CAFs are closely related to the tumor microenvironment (TME) of HCC and have been proven to influence HCC progression (13). Moreover, CAFs are now thought to be the primary driver of tumor growth due to complicated interactions with other cell types in the tumor microenvironment (14). Previous studies found CAFs undergo epigenetic modifications to create secreted factors, exosomes, and metabolites that regulate tumor angiogenesis, immunology, and metabolism in addition to producing extracellular matrix components that facilitate the function and formation of stroma tumor (15, 16). HCC-derived CAFs not only promote tumor cell malignancy (17–19) but also attract immune cells to allow the development of immunosuppressive phenotypes for immune escape (20, 21). Because of their putative prooncogenic functions, research on CAFs as a therapeutic target is very popular in recent years (14). However, the deeper prognostic value of CAF-related gene signatures in HCC is unknown.

This study analyzed the HCC data from TCGA database to investigate the role of CAF-related genes in HCC. Cluster analysis was used to identify clusters based on the expression pattern of CAF-related genes. Based on independent prognostic CAF-related genes, a CAF-related prognostic model was built for the prognostic prediction of HCC. To further assess the model prediction potential, the HCC data from ICGC database was utilized to verify the model. Next, risk score was combined with clinicopathological characteristics associated with prognosis to build a clinical risk model for prognostic prediction. Furthermore, for further evaluating the prognostic significance of CAF-related genes, the relationship between HCC patients with different risk scores and immune infiltration characteristics of TME, chemosensitivity, immunotherapy response, or difference in functional enrichment was investigated. To summarize, these findings suggest that CAF-related gene signatures may be exploited as possible prognostic markers in the search for novel treatments for patients suffering from HCC.

## Materials and Methods

### Data Collection

From TCGA database (https://portal.gdc.cancer.gov/), the RNA sequencing data including 50 normal liver samples and 374 HCC samples were acquired. The microarray data profiles of GSE25097, including 268 HCC samples and 243 adjacent non-tumor samples, were downloaded from the GEO database (https://www.ncbi.nlm.nih.gov/geo/). Another set of RNA sequencing data, including 232 HCC samples, was acquired from ICGC database (https://icgc.org/). Besides, from the TCGA and ICGC databases, the clinical information corresponding to HCC samples (374 HCC samples from TCGA and 232 HCC samples from ICGC) was downloaded. Duplicate samples and samples with missing follow-up information were not included in this study.

### Acquisition of CAF-Related Genes

From the Molecular Signature Database v7.5.1 (MSigDB), two gene sets related to CAF (MISHRA CARCINOMA ASSOCIATED FIBROBLAST UP and MISHRA CARCINOMA ASSOCIATED FIBROBLAST DN) were obtained. Another CAF gene set including 596 genes was acquired from a previous study (22). Then CAF-related genes were obtained after overlapping genes were filtered. GEO2R was utilized to screen DEGs (|log2(fold change)| >1, adjusted p-value <0.05) in GSE25097 (23). The “DESeq2” R package was utilized to screen DEGs (|log2(fold change)| >1 & adjusted p value <0.05) in TCGA (24).

### Cluster Analysis

The cluster analysis was conducted using the “ComplexHeatmap” R package (25). The cluster method was Euclidean and both rows and columns were clustered. Besides, the gene expression data is standardized by row-based Z-score. The K-M curve was utilized to observe the survival difference between the two clusters. The principal component analysis (PCA) was used to verify the accuracy of cluster analysis results.

### Establishment and Validation of the Prognostic Risk Score Model

First, the expression data of CAF-related genes were retrieved from the sequencing data of HCC samples in the TCGA. The clinical HCC information was then combined with those of CAF-related genes. In the next step, the DEGs in TCGA were intersected with the integrated CAF-related gene set to get CAF-related genes in TCGA, then the CAF-related genes in TCGA were verified with the DEGs in GSE25097, and finally, the verified CAF-related genes in TCGA were screened out. CAF-related genes in TCGA related to OS of HCC patients were mined by univariate Cox regression analysis (P < 0.05). To minimize overfitting, Least absolute shrinkage and selection operator (LASSO) logistic regression analysis (ten-fold cross-validation) was utilized to further screen key CAF-related prognostic genes. cBioPortal, a TCGA computational tool, was used to analyze the mutations and putative copy-number alterations as well as the correlation of genes in HCC samples in TCGA. Furthermore, multivariable Cox regression analyses identified genes showing independent prognostic relevance and developed a risk score model for predicting the prognosis of TCGA patients with HCC. Each sample’s risk score was computed according to risk score = Intercept (-0.38655353) + (-0.690788635)×ARHGAP11A + (0.681687351) × DLGAP5 + (0.131165649) × RFPL4B + (-0.241494247)×TOP2A + (0.626517272) × TTK.

The model was evaluated on the ICGC database to ensure its practicality. Furthermore, several test methodologies were used. Median risk scores were used as the basis for patient grouping. The K-M curve was utilized to assess survival differences. Moreover, the prognostic model’s practicality was validated using receiver operating characteristic (ROC), PCA, and a heat map of survival status combined with expression difference and risk score distribution in the independent prognostic genes.

### The Correlation of Risk Score With Clinicopathological Characteristics

First, according to the sample ID, corresponding clinicopathological characteristics were merged with each patients’ risk score. Shapiro-Wilk was used to test for normality. Comparisons of the distributions between two groups were performed using a Wilcoxon signed-rank test. To screen clinicopathological characteristics associated with prognosis, Univariate, and multivariate COX analyses were used. Differences were considered statistically significant when they showed P value < 0.05.

### Prognostic Nomogram Development

The “rms” and “survival” R packages were used to construct a prognostic nomogram. Moreover, calibration plot, DCA, and ROC curve were applied in assessing prediction capabilities of the nomogram.

### Estimation of Chemotherapeutic Drug Sensitivity

To calculate IC50 of chemotherapeutic drugs for each sample, the “pRRophetic” R package was used (26). Shapiro-Wilk was used to test for normality. Spearman’s rank correlation coefficient was here to evaluate the association between the two groups (risk score and IC50). Differences were considered statistically significant when they showed P-value < 0.05.

### Estimation of Immunotherapy Response

The 43 immune checkpoints were acquired from previous studies (27). Comparisons of the distributions between two groups were made by Wilcoxon signed-rank test. Correlation analysis was conducted by Pearson method. Differences were considered statistically significant when they showed P-value < 0.05.

### Immune Infiltration Analysis

To evaluate the relative abundance of immune cell types between high-risk and low-risk groups, CIBERSORT (28), TIMER (29), quanTIseq (30), and MCP-counter (31) was performed. Comparisons of the distributions between two groups were made by Wilcoxon signed-rank test. Correlation analysis was conducted by Pearson method. Differences were considered statistically significant when they showed P-value < 0.05.

### Functional Enrichment Analysis

The enrichment score of samples from low- and high-risk score groups was calculated by Gene Set Variation Analysis (GSVA) in the “GSVA” R package (32). The minimum and maximum gene sets are set to 5 and 5,000, respectively. The gene expression profile of the two risk samples was used to evaluate the related pathways and molecular mechanisms, and the reference gene sets“c2.cp.kegg.v7.4.symbols” were downloaded from the molecular signatures database (https://www.gsea-msigdb.org/gsea/msigdb). In the low-risk and high-risk groups, differentially enriching pathways (fold change>1.5 & p value <0.05) were screened using rank sum test. Besides, to perform the GO and KEGG enrichment analysis, the “clusterProfiler” R package was employed (33).

### Protein-Protein Interaction Network

In the low-risk and high-risk groups, DEGs (|log2(fold change)| >1 and adjusted p value <0.05) were screened using the “limma” R package (34). Through the STRING (https://string-db.org; version: 11.5), we obtained PPI network data (interaction score >0.9). Cytoscape (v 3.8.2) was utilized to build PPI network view and to screen out hub genes in the DEGs by degree method. To compare the distributions of the two groups, Wilcoxon signed-rank test was used. HCC patients in the TCGA were grouped into two expression groups (low and high), according to the median of hub genes’ expression value. Survival differences of the two groups were shown by K-M curves. Furthermore, independent hub genes showing prognosis relevance were screened by univariate and multivariate COX regression. To investigate the association of the expressions of independent prognostic hub genes with immune infiltration fraction levels, the immune infiltration score was calculated using ssGSEA algorithm in the “GSVA”R package (32, 35).

## Results

### Identification of CAF-Related Genes in HCC

**Figure 1:**
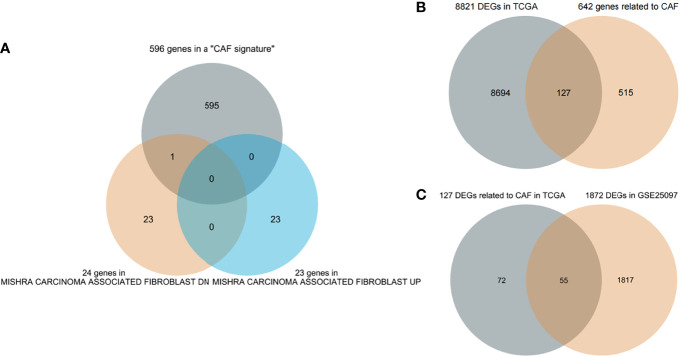
Screening genes **(A)** Three cancer-associated fibroblast (CAF)-related gene sets intersect to filter overlapping genes. **(B)** CAF-related DEGs in The Cancer Genome Atlas (TCGA). **(C)** Further validation in GSE25097.

**Figure 2:**
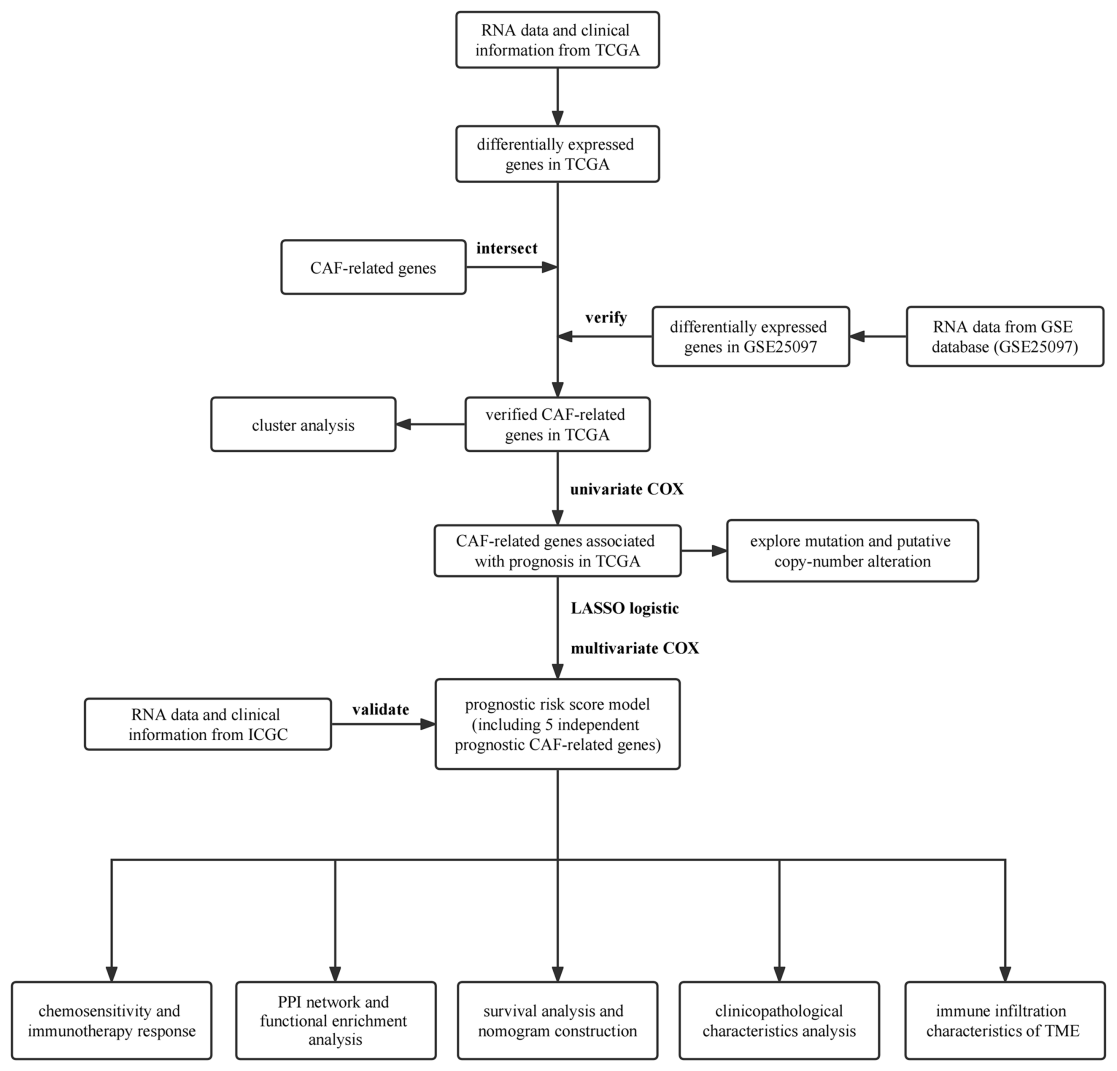
Flow chart.

First, 642 CAF-related genes were obtained by eliminating overlapping genes from the intersection of the three CAF-related gene sets (**Figure 1A**). From TCGA and GSE25097, 8,821 and 1,872 differentially expressed genes (DEGs) were mined by the “DESeq2” R packages and “GEO2R”, respectively. In the next step, 8,821 DEGs in TCGA and 642 CAF-related genes were intersected and 127 differentially expressed CAF-related genes were identified (**Figure 1B**). Finally, 127 CAF-related genes were verified by intersecting 1,872 DEGs in GSE25097 and 55 genes were filtered (**Figure 1C**). As depicted in **Supplementary Figure 1**, the expression of the 55 genes clearly differed between normal and tumor samples. **Figure 2** illustrates the flow of this study.

### Cluster Analysis of CAF-Related Genes in HCC

**Figure 3:**
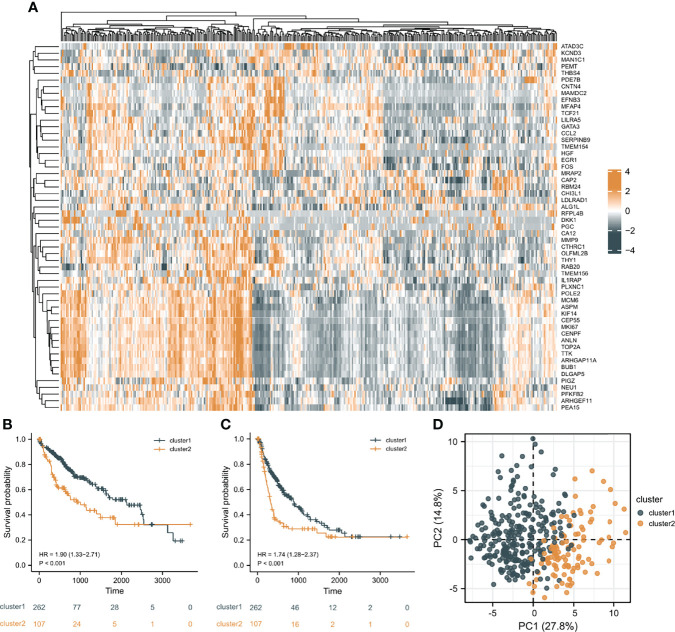
Cluster analysis results. **(A)** The heat map of the expression differences of 55 CAF-related DEGs in the two clusters. **(B)** The overall survival (OS) difference of the two clusters. **(C)** The progression free interval (PFI) difference of the two clusters. **(D)** Principal component analysis (PCA) plot.

In order to investigate the overall effect of the 55 CAF-related genes in HCC, cluster analysis was utilized to identify different clusters. As shown in the **Figure 3A**, it could be clearly observed that 369 samples were divided into two clusters (cluster 1 (n=262) and cluster 2 (n=107)) after the removal of one outlier sample. Subsequently, survival analysis was made to explore the survival difference between the two clusters. It was found that cluster 1 had a better OS and PFI than cluster 2, which was demonstrated in **Figures 3B, C**. Furthermore, PCA plot showed that the two clusters have a good resolution (**Figure 3D**), suggesting that this cluster pattern is feasible.

### Development of a Prognostic Model Based On Risk Score of HCC Patients

Prognostic genes were filtered from 55 differentially expressed CAF-related genes by Univariate Cox regression analysis and 22 genes were overall survival (OS)-related (P<0.05; **Supplementary Figure 2**). According to the 22 genes, the mutation and putative copy-number alterations profile of the CAF-related genes with prognostic relevance in HCC was described. Mutations and presumed copy-number changes of CAF-related genes were found in 175 of 366 HCC cases, with a frequency of 48% (**Supplementary Figure 3**). CTHRC1 had the highest mutation and putative copy-number alteration frequency than ARHGEF11. However, CEP55 exhibited few mutations and putative copy-number alterations in HCC samples. Besides, further analysis was performed to explore mutation mutual exclusivity and co-occurrence relationship. **Supplementary Figure 3** presents genes pairs with q-value <0.05 (derived from Benjamini-Hochberg FDR correction procedure) in the analysis results, which suggested that 19 pairs of genes with significant correlation only have a co-occurrence relationship, and ARHGEF11 was significantly positively correlated with most genes, including PEA15, OLFML2B, ASPM, KIF14, CENPF, and RAB20. Then, using LASSO Cox regression, 14 prognostic gene signatures were identified from the 22 CAF-related genes (**Supplementary Figure 4A, B**). **Supplementary Figure 4C** depicts the 14-gene correlation network. Finally, five genes (ARHGAP11A, DLGAP5, RFPL4B, TOP2A, TTK) with independent prognostic relevance were identified by multivariate Cox regression and were utilized to build a prognostic risk score model (**Supplementary Figure 4D**).

**Figure 4:**
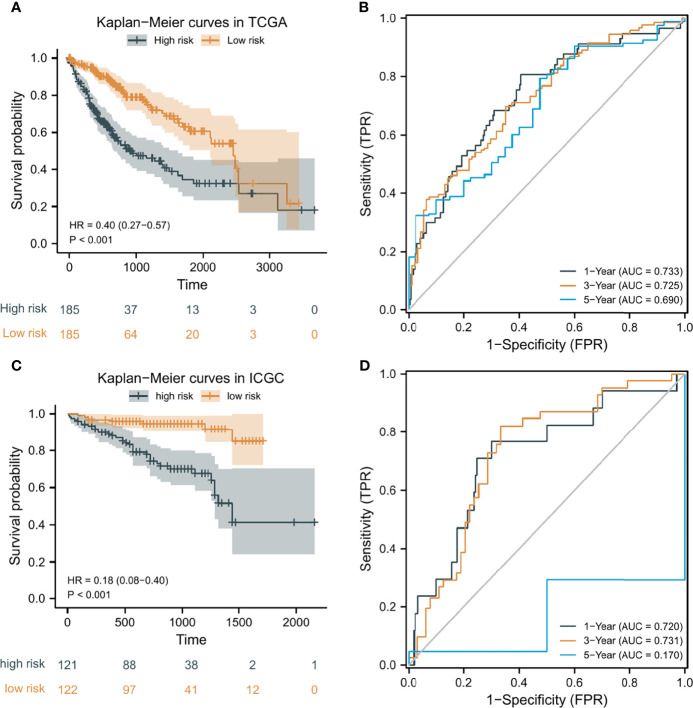
Kaplan-Meier (K-M) curves and time-dependent receiver operating characteristic (ROC) curves of the prognostic model of HCC patients. **(A, C)** K-M curves of the prognostic model in TCGA and ICGC, respectively. **(B, D)** 1-year, 3-year, and 5-year ROC curves of the prognostic model in TCGA and ICGC, respectively.

**Figure 5:**
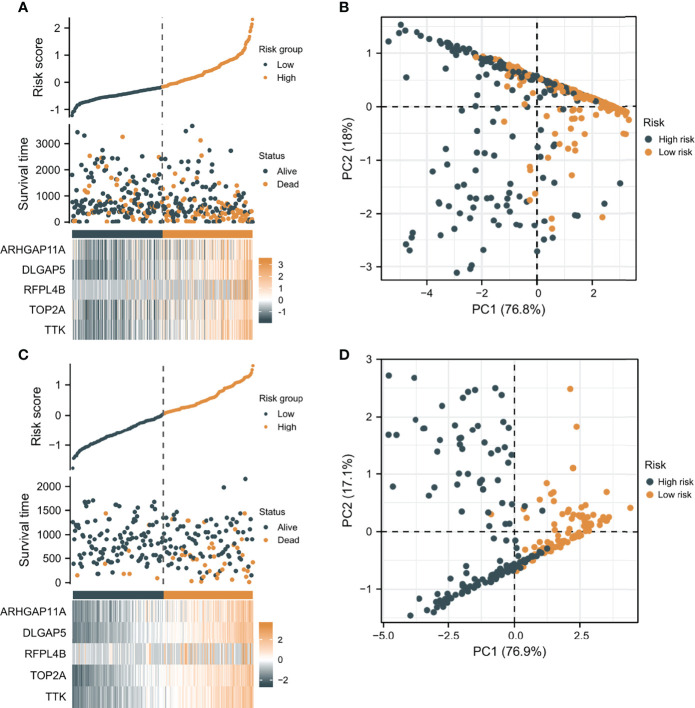
The verification of the prognostic model’s predicting power. **(A)** The heat map combined with survival status, risk score distribution, and expression difference in the 5 independent prognostic genes in TCGA. **(B)** PCA based on the 5 independent prognostic genes in TCGA. **(C)** The heat map combined with survival status, risk score distribution, and expression difference in the 5 independent prognostic genes in ICGC. **(D)** PCA of the 5 independent prognostic genes in ICGC.

According to the median risk score as the cutoff value, the patients were sorted and grouped into two groups: high-risk (n = 185) and low-risk (n = 185). In TCGA, compared with the low-risk score group, the high-risk score group showed a shorter OS (P<0.001; **Figure 4A**). To the reliability of the risk score and the model, we plotted a time-dependent ROC curve, see **Figure 4B** for the areas under the curve (AUCs). Furthermore, the heat map of survival status combined with expression difference and risk score distribution in the 5 independent prognostic genes, and PCA, were utilized to differentiate individuals with distinct risk levels. As shown in **Figures 5A, B**, there is a high degree of discrimination between the high-risk and low-risk scores groups.

### Verifying the Prediction Capability of the Prognostic Model

HCC samples from ICGC were categorized into low-risk and high-risk score groups to verify the prognostic risk score model, according to the median risk score. As the same results in TCGA, a shorter OS was observed in the high-risk score group (P<0.001) than in the low-risk score group in ICGC (**Figure 4C**), demonstrating that the predicting model built on risk score in TCGA had a good ability to predict OS. The AUCs were presented in **Figure 4D**. The risk model constructed based on the ICGC database was further tested using a heat map of survival status along with risk score distribution and expression difference in the 5 independent prognostic genes and PCA. As shown in **Figures 5C, D**, the prognostic model in ICGC had a strong capacity for predicting prognosis.

### The Correlation of Clinicopathological Characteristics With Risk Score

The distribution of risk scores in corresponding samples was investigated in terms of age, gender, clinical stage, histological grade, and AFP level. Higher risk scores were associated with a higher pathologic stage (P = 0.003), histological grade (P < 0.001), and AFP level (P < 0.001) (**Supplementary Figures 5C–E**), but not with age or gender (P > 0.05; **Supplementary Figures 5A, B**). Univariate and multivariate Cox analysis included the following factors: age, gender, pathological stage, histological grade, AFP level, and prognostic risk score. The findings suggested that pathologic stage, gender, prognostic risk score, and age were factors related to prognosis (P < 0.05), and that only prognostic risk score, pathologic stage, and age were factors with independent prognosis relevance (P < 0.05; **Supplementary Figures 6A, B**).

### Establishment of Prognostic Nomogram

**Figure 6:**
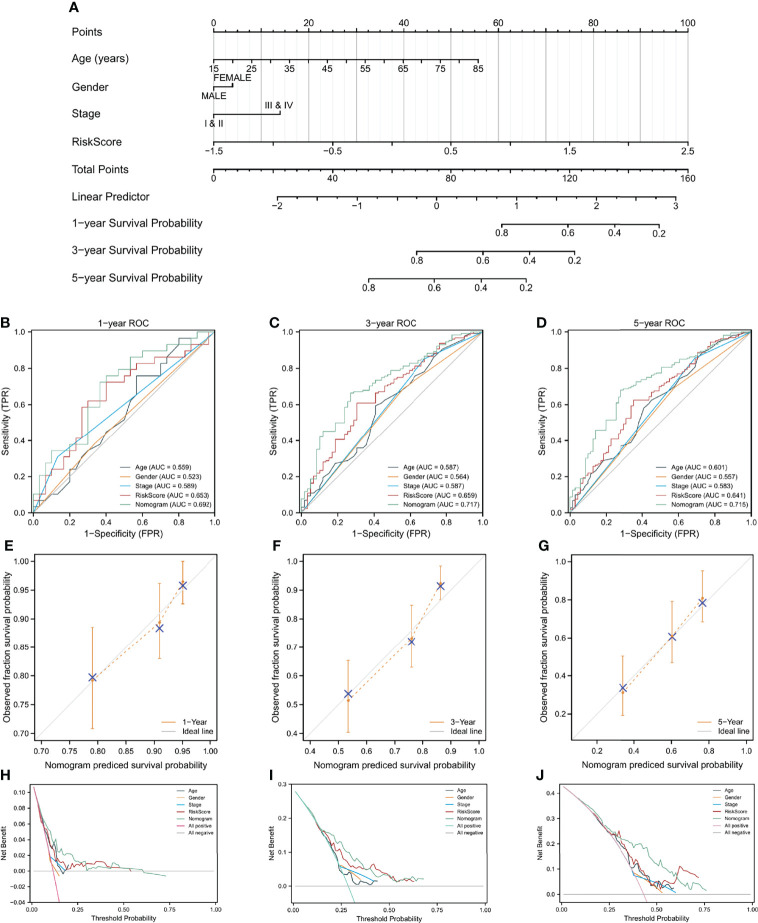
A nomogram build on CAF-related risk score and clinicopathological characteristics related to prognosis to predict OS of HCC patients from TCGA and the verification of its accuracy. **(A)** The nomogram for predicting 1-year, 3-year, and 5-year OS of HCC patients. **(B–D)** ROC curves of the nomogram and other indicators including age, gender, stage, and risk score. **(E–G)** The calibration plots of the nomogram. **(H–J)** Decision curve analysis for evaluating the accuracy of the nomogram to predict 1-year, 3-year and 5 year OS of individuals with HCC.

The prognostic risk score was combined with age, gender, and pathologic stage to build a nomogram for OS prediction (**Figure 6A**). **Figures 6B–D** depicted the ROC curves of multiple indicators and demonstrated that the nomogram had a stronger predictive capacity than any other indicator. As illustrated in **Figures 6E–G**, the nomogram was predictive of the OS for HCC patients and demonstrated comparatively high accuracy, as shown by the calibration curves. Furthermore, decision curve analysis (DCA) revealed that the nomogram outperformed a single independent predictive parameter (**Figures 6H–J**). To summarize, the predictive potential of the prognostic nomogram was validated from several angles.

### Prediction of Chemotherapeutic Drug Sensitivity

**Figure 7:**
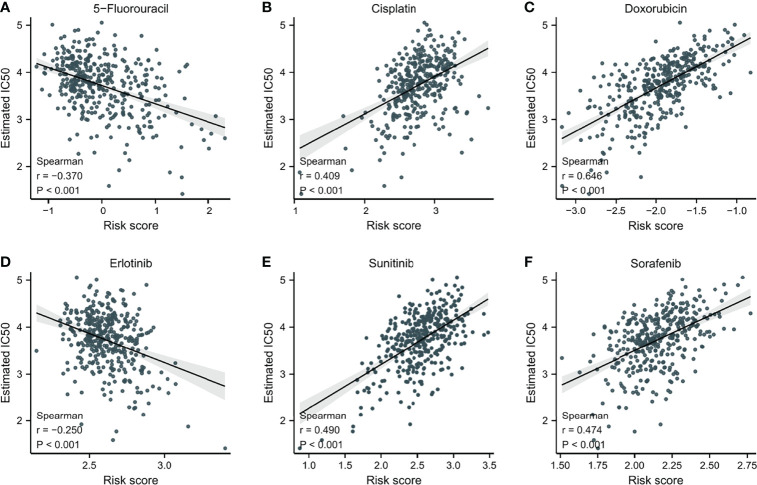
The correlation between IC50 of different drugs and risk score in HCC patients. **(A)** 5-Fluorouracil. **(B)** Cisplatin. **(C)** Doxorubicin. **(D)** Erlotinib. **(E)** Sunitinib. **(F)** Sorafenib.

To explore potential novel therapies for HCC, the correlation between risk score and chemosensitivity was analyzed. ESMO Clinical Practice Guidelines for HCC (36) was used as a reference to get common chemotherapeutic drugs. The “pRRophetic” R package calculated half-maximal inhibitory concentration (IC50) for predicting the correlation of chemosensitivity between different risk score samples in TCGA. The correlation between the sensitivity to six predicable chemotherapeutic drugs and different risk scores was presented in **Figure 7**. Low-risk score samples showed higher sensitivity to cisplatin, doxorubicin, sunitinib, and sorafenib (**Figures 7B, C, E, F**); P<0.001), while high-risk score samples showed higher sensitivity to 5-Fluorouracil and Erlotinib (**Figures 7A, D**); P<0.001).

### Immune Infiltration Characteristics of TME Between Low-Risk and High-Risk Groups

**Figure 8:**
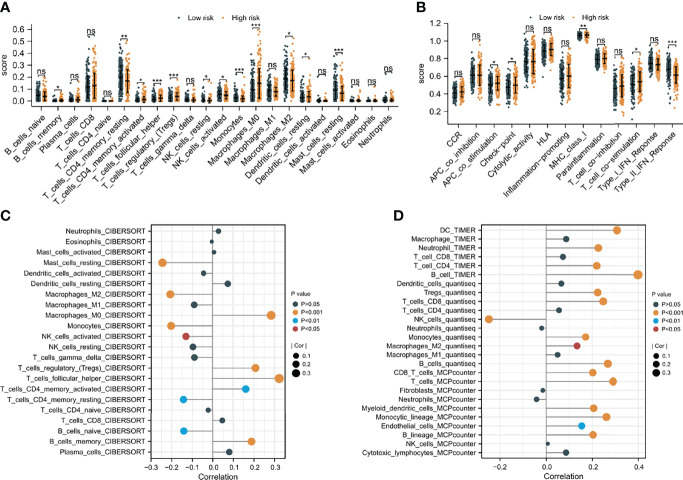
The difference in immune infiltration characteristics of tumor microenvironment between low and high risk groups. **(A)** Immune cell infiltration analysis **(B)** immune-related pathways infiltration analysis **(C, D)** the correlation analysis of immune cells by various algorithms statistical significance is indicated by the following symbols: ns, *p* ≥ 0.05; *, *p* < 0.05; **, *p* < 0.01; ***, *p* < 0.001.

Since CAFs are closely associated with TME (13), an investigation was made to explore the difference in immune infiltration characteristics of TME between the two risk groups. As shown in **Figures 8A, B**, 11 of the 22 immune cells and 5 of the 13 immune-related pathways were found with a significant difference in infiltrating fraction between distinct risk levels group. Furthermore, to explore the correlation between risk score and immune infiltration score, immune analysis based on various algorithms (CIBERSORT, TIMER, quanTIseq, and MCP-counter) were performed. As shown in **Figures 8C, D**, 20 microenvironment components were positively correlated with risk scores, while 7 microenvironment components were negatively related to risk scores. B cells, CD8 T cells, and Tregs were found with a significantly positive correlation with risk scores, which were confirmed by more than one immune analysis algorithm (**Figures 8C, D**). To further explore the correlation between immune infiltration characteristics and various risk score groups, ESTIMATE was used to calculate TME scores and tumor purity. The results showed that the stromal score was found to have a significantly higher level in the low-risk group (**Supplementary Figure 7**). These findings suggested risk score based on CAFs correlated with tumor immune microenvironment.

### Prediction of Immunotherapy Response

Recently, immunotherapy for hepatocellular carcinoma has been a new hot research topic, and immune checkpoint inhibitors (ICIs) are at the forefront of this revolution (37). To explore the correlation of CAF-related signatures with immunotherapy response, the expression of immune checkpoints between different risk score groups was compared. As **Supplementary Figure 8A** showed, the expression of 32 of 43 immune checkpoints was found with a significant difference between low- and high-risk groups, and interestingly, all the 32 immune checkpoints were highly expressed in the high-risk group. In addition, as classical representatives of immune checkpoints, the expression of PD-1 and CTLA-4 were found to have a significantly positive correlation with risk scores (**Supplementary Figures 8B, C**). In short, these results indicated that patients with high-risk scores are more likely to benefit from treatment with ICIs.

### Functional Enrichment Analysis

To investigate pathways and molecular processes between the two risk groups, gene set variation analysis (GSVA) enrichment was utilized. As demonstrated in **Supplementary Figure 9**, a wide range of molecular biological processes showed enrichment in the high-risk group, whereas the majority of metabolic pathways were enriched in the low-risk group.

**Figure 9:**
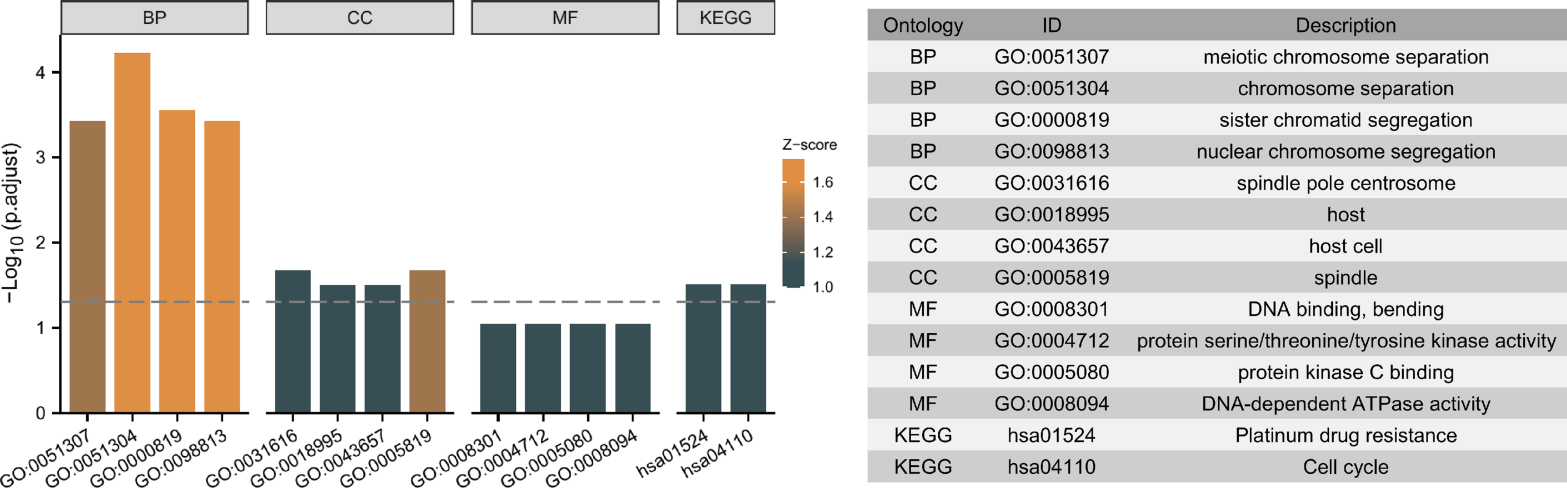
Top ten representative pathways related to the 5 independent prognostic genes in GO and KEGG enrichment results.

Moreover, the 5 independent prognostic genes were subjected to Gene Ontology (GO) and Kyoto Encyclopedia of Genes and Genomes (KEGG) enrichment analysis to explore possible pathways and molecular functions. **Figure 9** shows that the most highly enriched biological processes were meiotic chromosome separation, chromosome separation, sister chromatid segregation, and nuclear chromosome segregation. Additionally, KEGG enrichment analysis indicates that the 5 genes might participate in pathways including platinum drug resistance and cell cycle (**Figure 9**).

### Construction of PPI Network Based on DEGs Between the Low-Risk and High-Risk Groups

**Figure 10:**
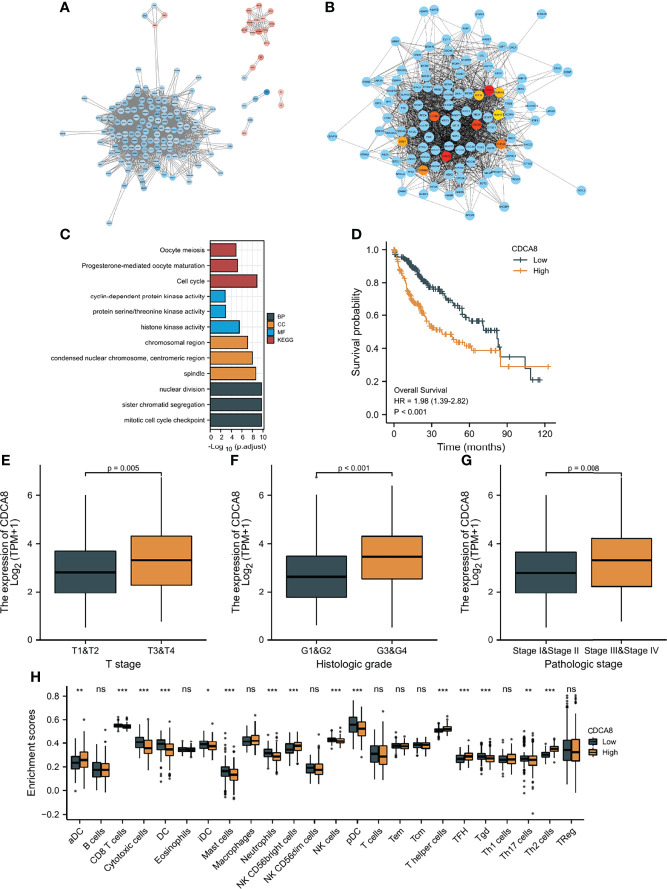
Protein-protein interaction (PPI) analysis. **(A)** PPI network constructed using Cytoscape; Blue: high expression DEGs in high-risk group, red: high expression DEGs in low-risk group. **(B)** Top ten hub genes screen by degree method. **(C)** GO and KEGG analyses of top ten hub genes. **(D)** K-M curves of CDCA8 expression level. **(E–G)** The relationship between CDCA8 expression level and clinicopathological characteristics. **(H)** The difference in immune infiltration fraction between low and high expression groups of CDCA8 statistical significance is indicated by the following symbols: ns, *p* ≥ 0.05; *, *p* < 0.05; **, *p* < 0.01; ***, *p* < 0.001.

First, the “limma” R package screened DEGs between the low-risk and high-risk groups. STRING was utilized to develop a PPI network based on the DEGs. The PPI network was visualized using Cytoscape (**Figure 10A**), and hub genes were identified by the degree method (**Figure 10B**). CDK1, AURKB, CDC20, BUB1, KIF11, CCNB1, TOP2A, CDCA8, BUB1B, and CCNA2 were identified as the top ten hub genes. All ten genes were significantly higher-expressed in tumor samples than in normal samples. To explore the differences in survival, patients were grouped into low- and high-expression groups by the median expression of the ten genes. Except for BUB1B, the K-M curves show that the low expression group showed a longer OS (**Figure 10D** and **Supplementary Figures 10A–I**). Furthermore, biological pathways and progress related to the ten genes were identified by GO and KEGG enrichment analyses. According to the findings of KEGG enrichment analyses, ten genes were enriched in cell cycle, progesterone-mediated oocyte maturation and oocyte meiosis. In addition, the ten genes were associated with pathways such as condensed nuclear chromosome, protein serine/threonine kinase activity, mitotic cell cycle checkpoint, cyclin-dependent protein kinase activity, histone kinase activity, chromosomal region, spindle, and centromeric region, sister chromatid segregation, and nuclear division, according to the results of GO enrichment analyses (**Figure 10C**). To identify the genes with independent prognosis relevance, univariate and multivariate COX regression were used. Only CDCA8 and TOP2A were found to be independent prognostic genes (**Supplementary Figures 10J, K**). Since TOPA2 was identified as a CAF-related independent prognostic gene and its role in HCC was explored above, next only the role of CDCA8 in HCC was further investigated. As shown in **Figures 10E–G**, high CDCA8 expression is associated with a high T stage, histological stage, and pathological stage, indicating that up-regulated expression of CDCA8 is related to a highly malignant HCC. In addition, the association of CDCA8 expression level with immune cell infiltration level was studied by immune cell infiltration analysis. As shown in **Figure 10H**, patients with low CDCA8 expression have a high infiltration of cytotoxic and NK cells.

## Discussion

Increasing evidence has shown that CAFs are crucial players in HCC progression (15). For example, CAFs play an important role in the multicellular, stromal-dependent changes that contribute to HCC development. A previous study indicated that CAF-mediated cellular crosstalk supports HCC progression (9). The Yugawa et al. study suggested that CAFs enhance HCC development by downregulating exosomal miR-150-3p (38). Moreover, research by Chen et al. demonstrated that CAFs activate M2-polarized macrophages to promote HCC development through the plasminogen activator inhibitor-1 pathway (12). It is proven that the BAFF/NFκB axis in CAFs leads to sorafenib-resistant HCC cells (39). However, most research concentrates on the impact of a single CAF-related gene regulator in HCC and the combined effects of numerous CAF-related genes remain unclear. The investigation of the involvement of CAF-related gene signatures in HCC might aid in the understanding of CAFs in HCC development, hence, directing to an appropriate treatment strategy.

This is the first research probing into the prognostic significance of CAF-related gene signatures for HCC patients. To improve the reliability of the current results, the GEO database was used to screen CAF-related gene sets in TCGA and the potential of the prognostic model built using CAF-related genes was validated by the ICGC database. Moreover, for obtaining representative prognostic genes and increasing the applicability of the model, LASSO Cox regression and multivariate Cox regression analyses were conducted to screen core CAF-related genes with independent prognosis relevance and to construct a risk score prognostic model. Among the 5 CAF-related genes with independent prognosis relevance in TCGA, the majority of genes played roles in cancer progression. Previous research linked the overexpression of ARHGAP11A, DLGAP5, TOP2A, and TTK to the development of HCC (40–43). ARHGAP11A is a gene that encodes for a protein regulating cell cycle-dependent motility. Recently, ARHGAP11A has been shown to enhance malignant HCC development through an ARHGAP11A-Rac1B interaction (40). Moreover, the Lu et al. study demonstrated that HOXD-AS1 upregulated ARHGAP11A resulting in induced metastasis *via* competitively binding to microRNA-19a (44). However, the function of ARHGAP11A in CAFs is unknown. This study included ARHGAP11A in the CAF-related genes prognostic model to investigate the role of ARHGAP11A in CAFs.

Moreover, DLGAP5 is a microtubule-associated protein and mitotic phosphorylated substrate of Aurora-A. The Liao et al. research suggested that methylation negatively regulated DLGAP5 expression, which indicates that DLGAP5 may be a methylation biomarker in HCC (41). In a previous study (45), DNA topoisomerase 2-alpha (TOP2A) has been identified as a core gene in HCC, which was again proved by this study. TOP2A, a m6A RNA methylation-modified gene, plays an important function in controlling DNA double strand unwinding and has been therefore regarded as a therapeutic target (45). TOP2A, like DLGAP5, may also be a methylation biomarker in HCC. Monopolar spindle 1 (TTK) is a gene that encodes a dual serine/threonine and tyrosine protein kinase and is required for chromosome alignment and the spindle assembly checkpoint (43). Prior research found that *via* the activation of the Akt/mTOR and MDM2/p53 signaling pathways, TTK stimulates migration and cell proliferation in HCC (46). In addition, some studies indicated that DLGAP5, TOP2A, and TTK were related to the prognosis of individuals with HCC (43, 47, 48). This study verified these previous conclusions again and evaluated these genes in CAFs in terms of their prognostic significance.

The prognostic risk score model developed with the 5 CAF-related genes was predictive of the OS of patients suffering from HCC. We found that the low-risk group had a longer OS than the high-risk group in the TCGA, which has been confirmed in ICGC database, suggesting that the prognostic model had a strong performance to predict the populace with poor OS in HCC. In addition, the prognostic risk score model combined with clinicopathological characteristics associated with prognosis increased the model’s predictive capability and clinical applicability.

To better understand the impact of the risk score model on HCC, chemosensitivity differences among patients with two risk scores (low, high) were explored. Cisplatin and doxorubicin are widely used in the intra-arterial administration of chemotherapy in early and intermediate HCC (36). The higher sensitivity to cisplatin and doxorubicin in lower risk score patients demonstrated that the prognostic model had the ability to screen patients with relatively high sensitivity to cisplatin and doxorubicin to improve the efficacy in early and intermediate HCC. In the recent decade, sorafenib was recognized as the only available standard of care for advanced HCC (49). The chemosensitivity to sorafenib was negatively related to the risk score of HCC patients, which indicated the model could identify individuals with relatively higher sensitivity to sorafenib in advanced HCC, thus improving their prognosis.

Recently, TME is considered to be an important part of the occurrence, development, invasion and metastasis of HCC (50). A variety of immune or stromal cell types observed in HCC collaborate in the formation of an immunosuppressive TME and their presence is often associated with prognosis (51), which was also observed in this study. For instance, there was also a significant difference in stromal scores between the low-risk and high-risk groups with significantly different OS. A previous study discovered that CAFs were related to the suppression of NK cell activity in HCC (13), but the link between CAFs and other numerous immune cells in HCC is uncertain. In this research, patients scored as having a high risk showed a greater infiltration of B cells, CD8 T cells, and immunosuppressive cells such as Tregs. These findings might help with personalized immunotherapy and improvement of the treatment outcomes.

This study may have several limitations. First, the lack of in-depth mechanism research is the main limitation. Therefore, experimental research must be conducted to confirm the detailed molecular processes of the CAF-related gene signatures in the future. Secondly, further clinical research should be carried out to verify the prognostic model built on the 5 CAF-related genes. Furthermore, the study relied solely on public sources data, which may involve selection bias.

## Conclusion

In conclusion, this study evaluated the prognostic value of CAF-related gene signatures in HCC. Based on the expression pattern of CAF-related genes, two clusters were identified and found to have a difference in OS and PFI. CAF-related prognostic risk score model can be utilized for prognostic prediction of HCC. The risk score based on CAFs can predict chemotherapy sensitivity and immunotherapy response for improving prognosis in HCC. Additionally, there was a correlation between immune infiltration characteristics of TME and patients with different risk levels, which may aid in creating a cooperative effect in CAF-targeted treatments and immunotherapy. These findings present a favorable predicting model in prognosis, potentially paving the way for individualized HCC therapy in the future.

## Data Availability Statement

Publicly available datasets were analyzed in this study. This data can be found here: GEO (https://www.ncbi.nlm.nih.gov/geo), TCGA (https://cancergenome.nih.gov), and ICGC (https://dcc.icgc.org) databases.

## Ethics Statement

All data used in this study were from public databases, therefore ethical approval was not required.

## Author Contributions

Conception and design: WD. Collection and collation of data: WD, YX, and HH. Data analysis and interpretation: WD, YX and HH. Manuscript writing and revisions: WD, YX, and HH. Final approval of manuscript: All authors. Accountable of all aspects of work: All authors.

## Conflict of Interest

The authors declare that the research was conducted in the absence of any commercial or financial relationships that could be construed as a potential conflict of interest.

## Publisher’s Note

All claims expressed in this article are solely those of the authors and do not necessarily represent those of their affiliated organizations, or those of the publisher, the editors and the reviewers. Any product that may be evaluated in this article, or claim that may be made by its manufacturer, is not guaranteed or endorsed by the publisher.
