# Supplementary figures and images for: Prognostic Value of Cancer-Associated Fibroblast-Related Gene Signatures in Hepatocellular Carcinoma
Source: Front Endocrinol (Lausanne). 2022 Jun 6;13:884777. doi: 10.3389/fendo.2022.884777 (PMC9207215; doi:10.3389/fendo.2022.884777)

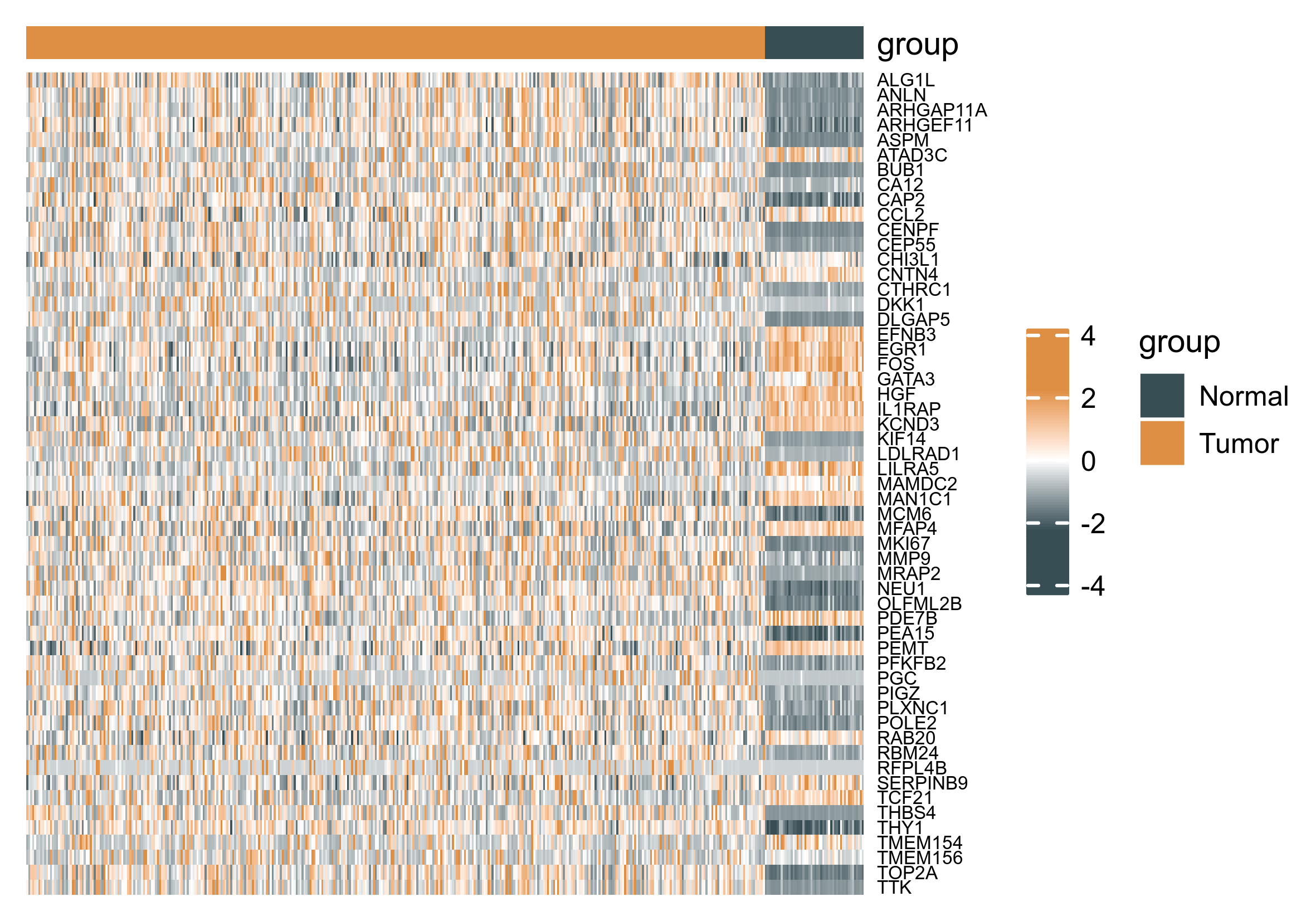

Supplement: Supplementary file 1 [file Image_1.tiff]

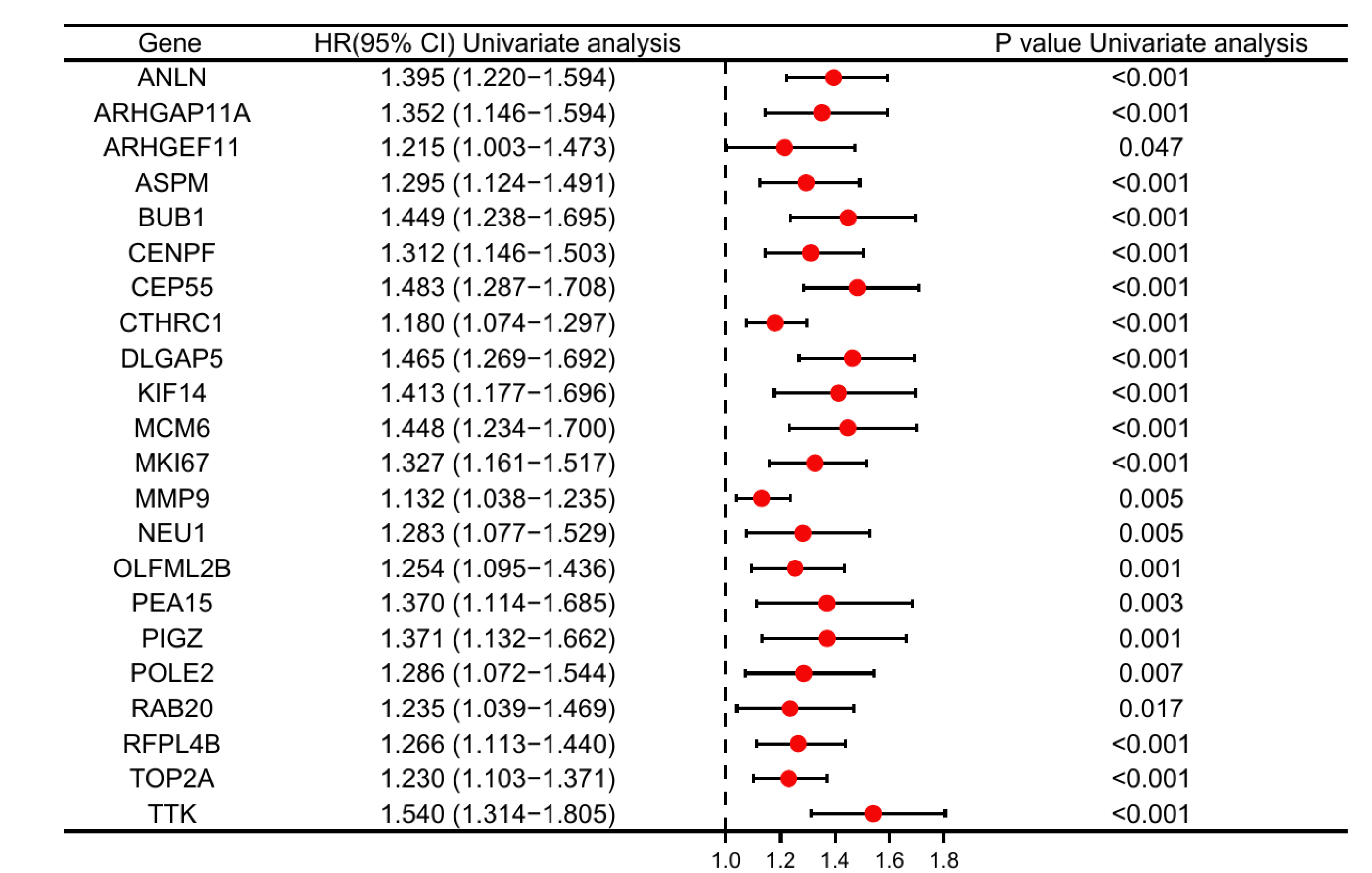

Supplement: Supplementary file 2 [file Image_2.tiff]

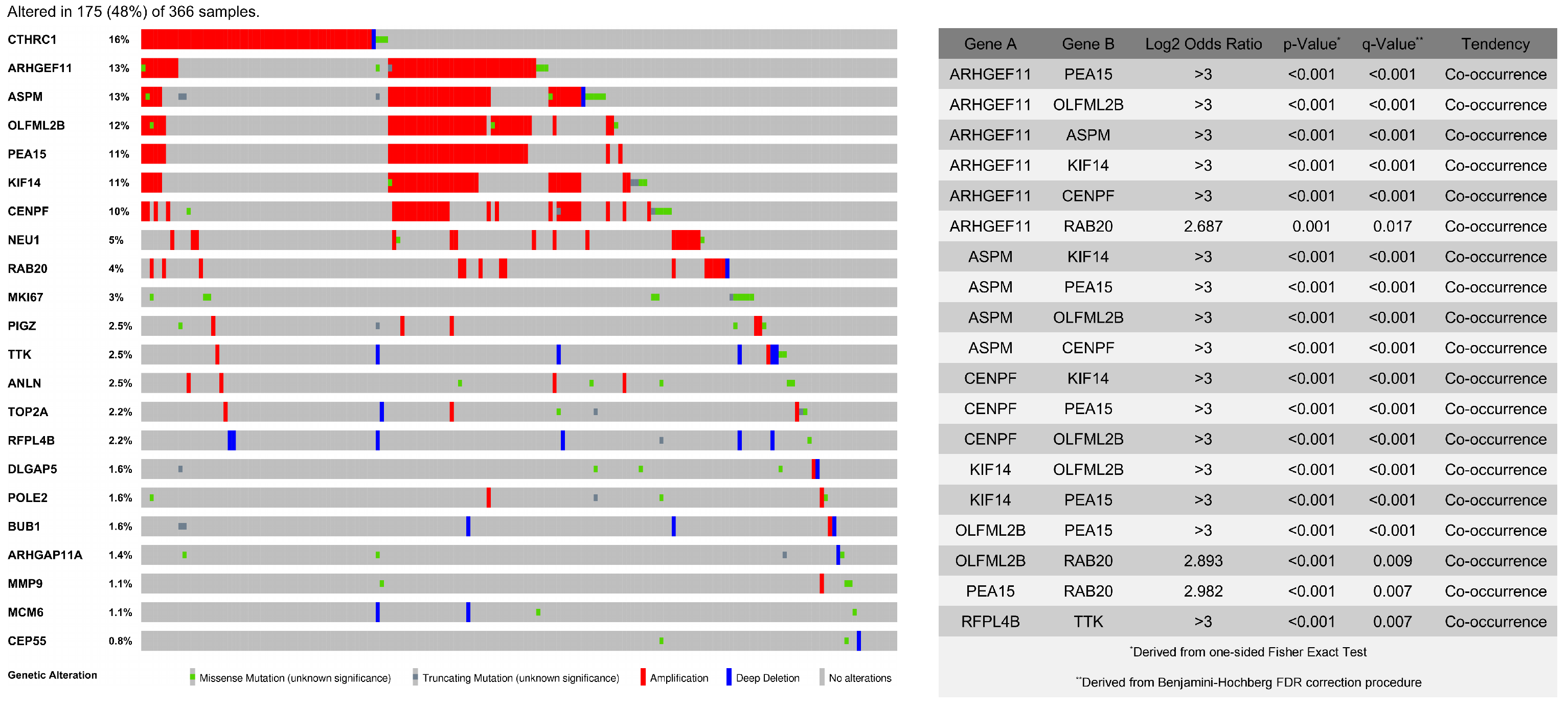

Supplement: Supplementary file 3 [file Image_3.tiff]

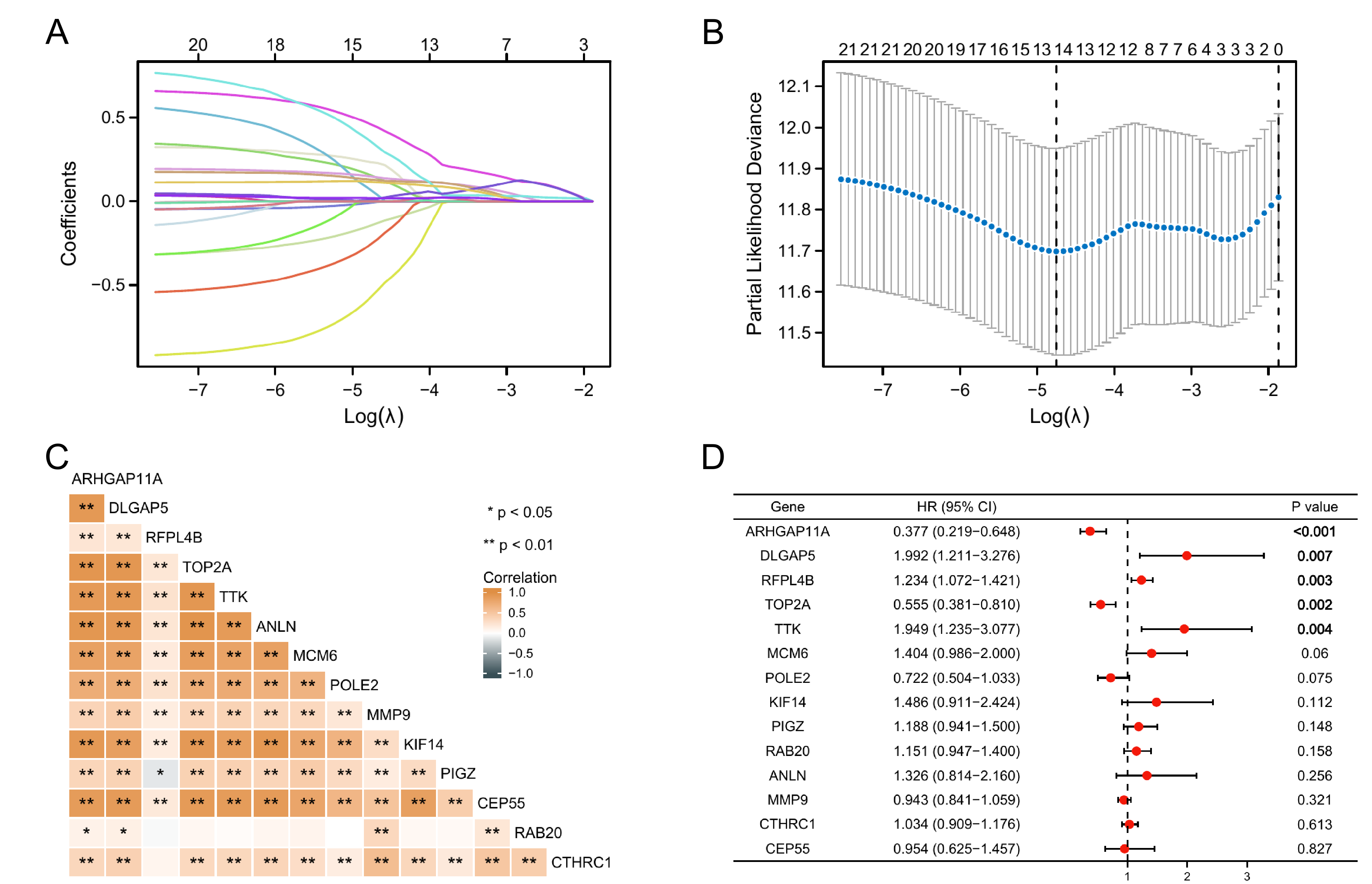

Supplement: Supplementary file 4 [file Image_4.tiff]

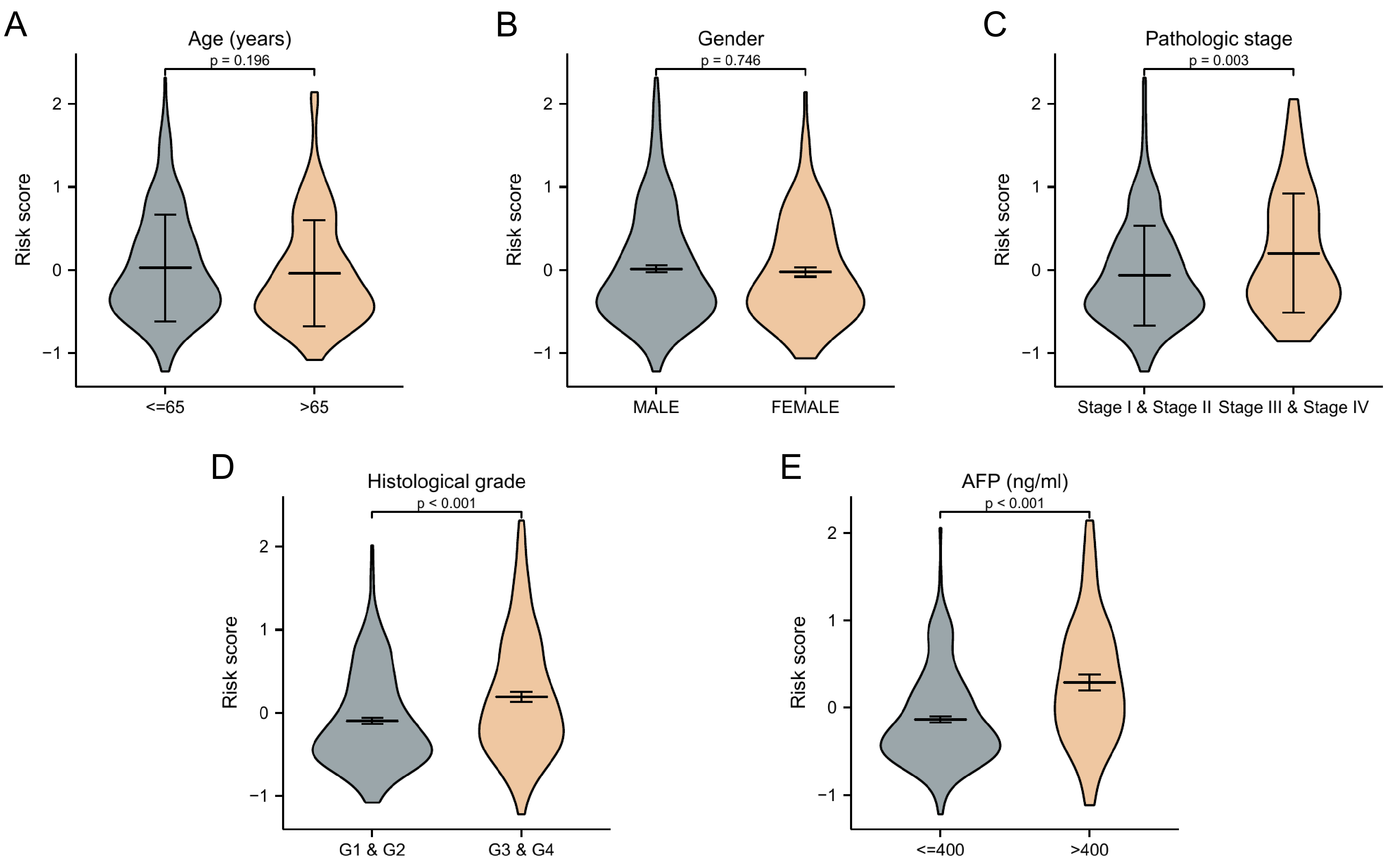

Supplement: Supplementary file 5 [file Image_5.tiff]

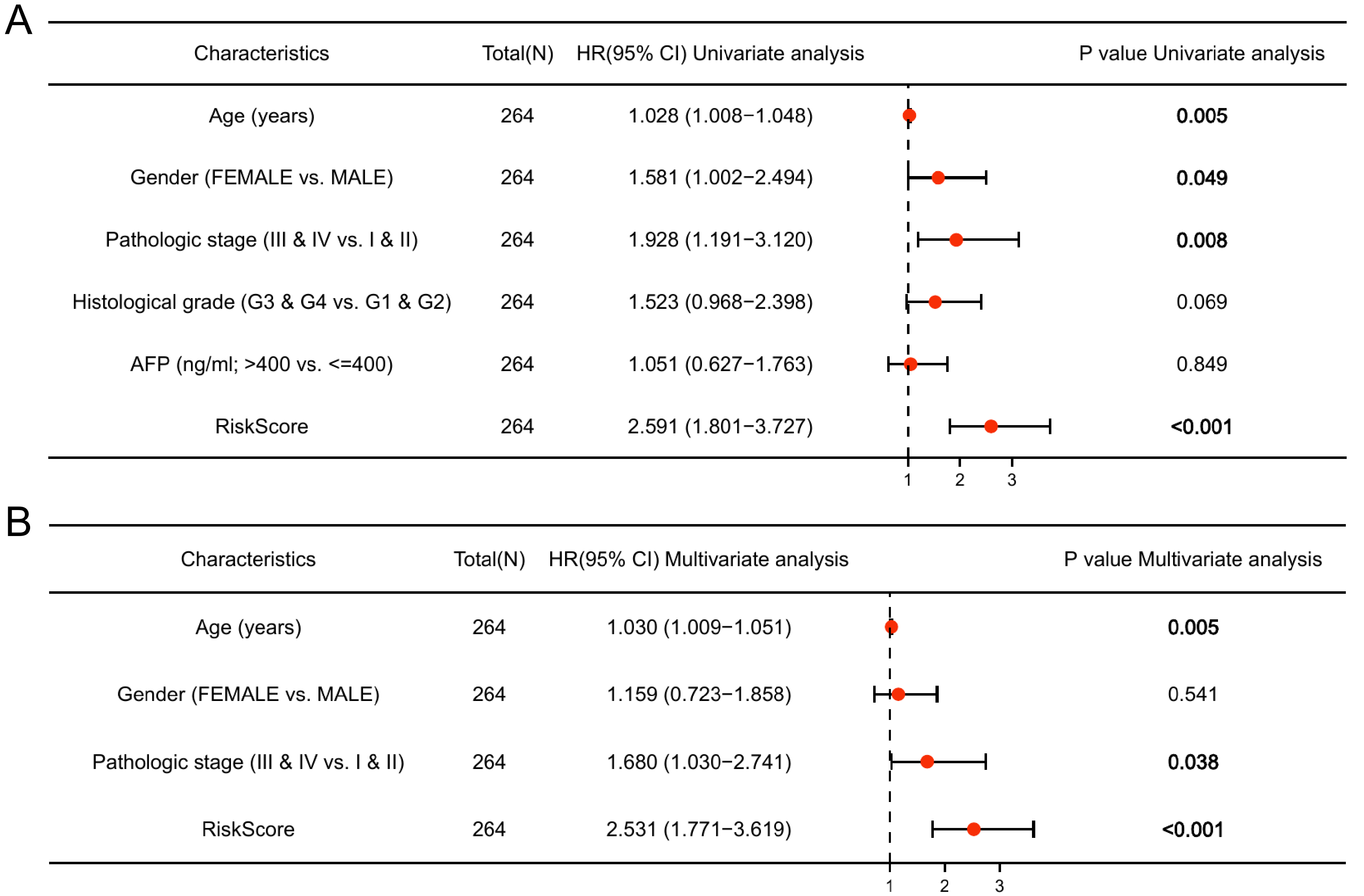

Supplement: Supplementary file 6 [file Image_6.tiff]

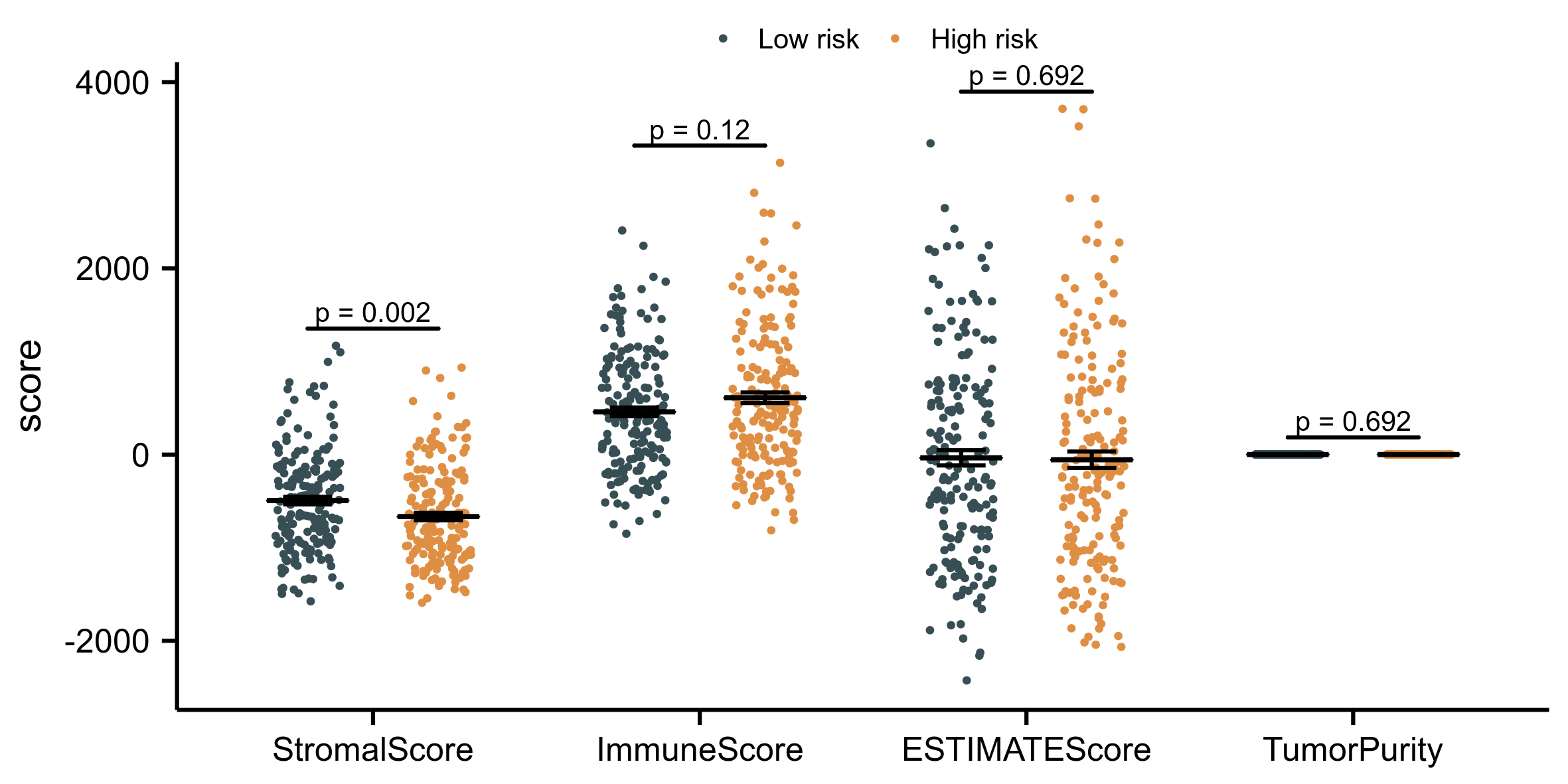

Supplement: Supplementary file 7 [file Image_7.tiff]

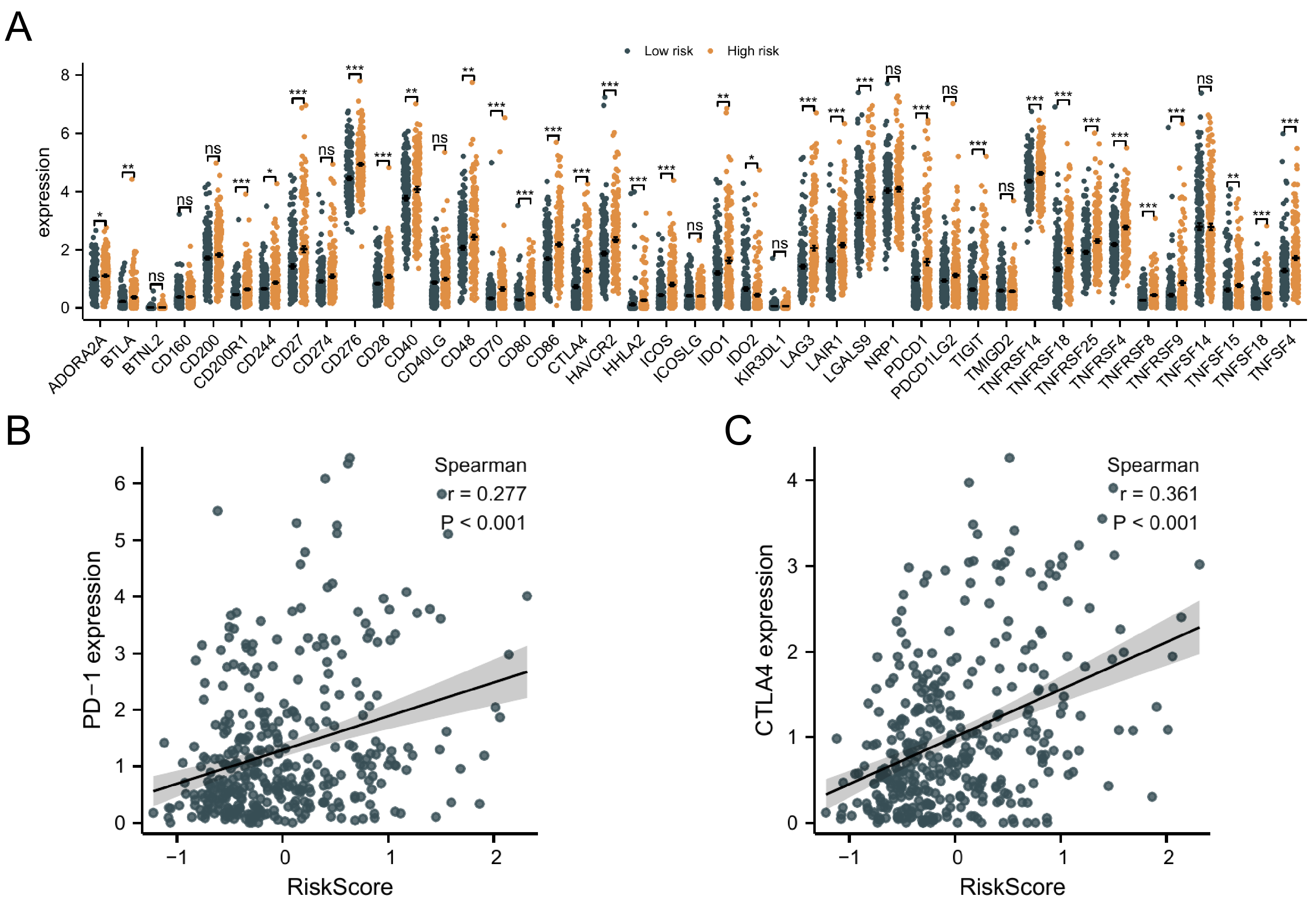

Supplement: Supplementary file 8 [file Image_8.tiff]

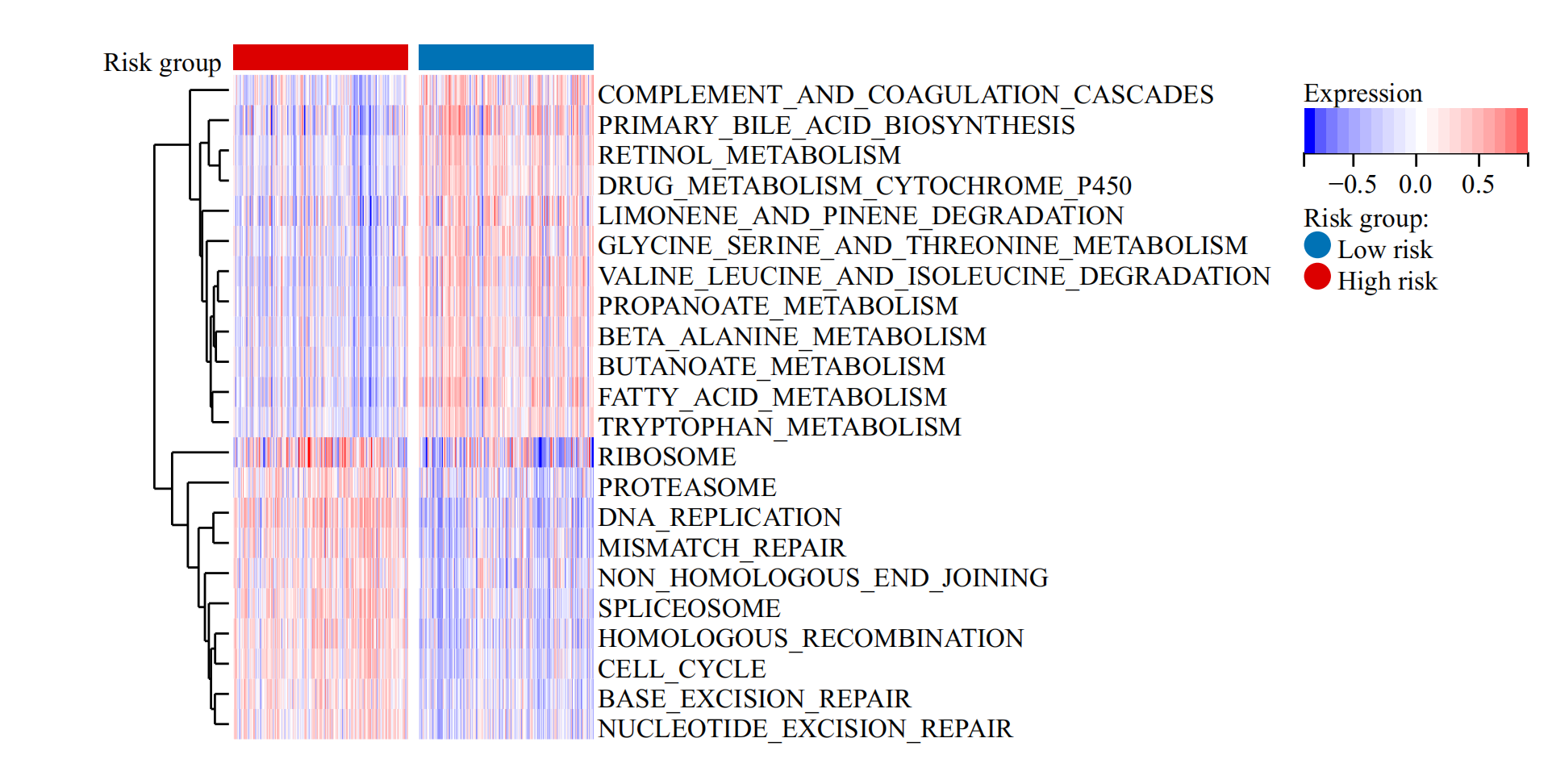

Supplement: Supplementary file 9 [file Image_9.tif]

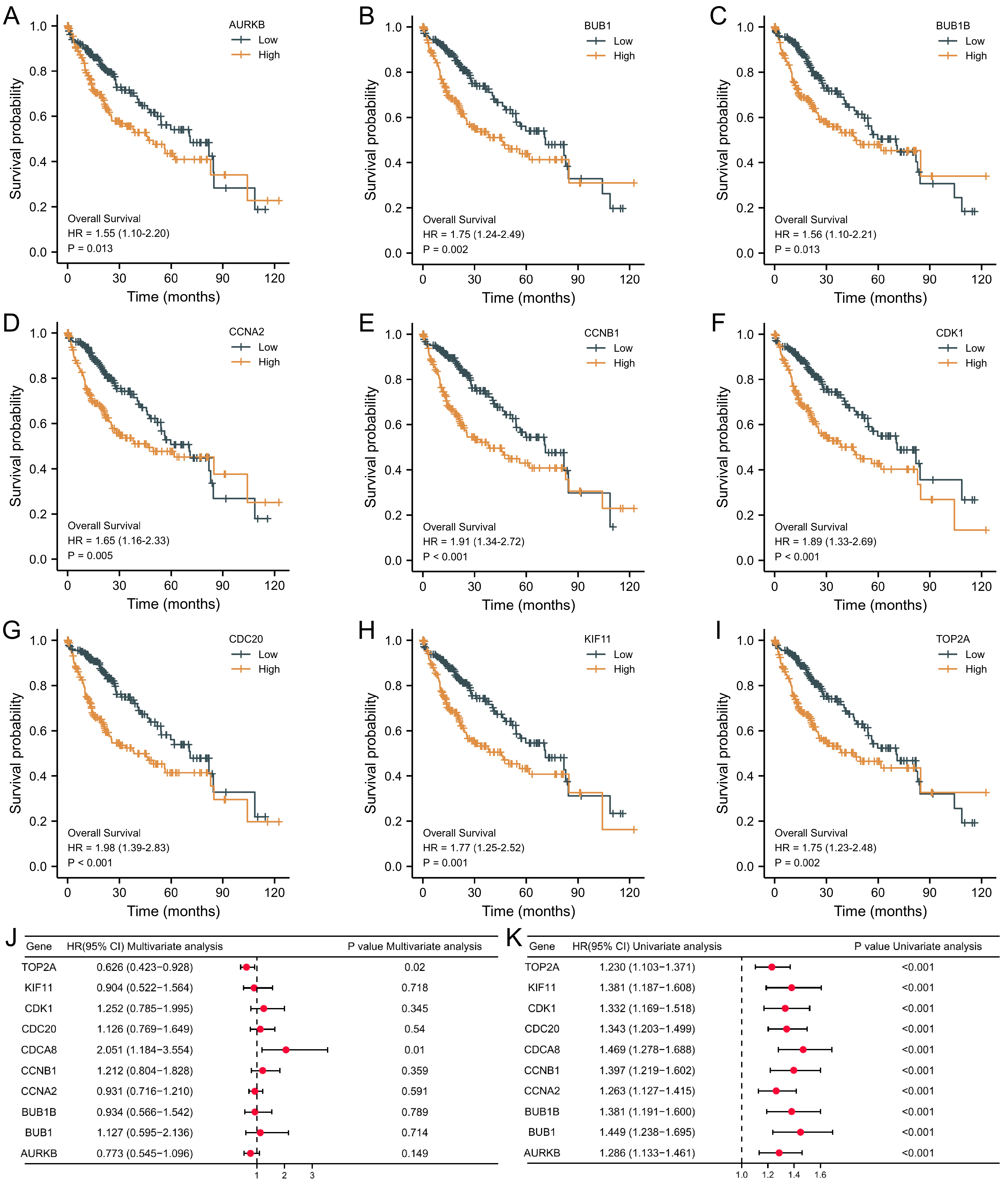

Supplement: Supplementary file 10 [file Image_10.tiff]
