# Supplementary material for: Prognostic Value of Cancer-Associated Fibroblast-Related Gene Signatures in Hepatocellular Carcinoma
Source: Front Endocrinol (Lausanne). 2022 Jun 6;13:884777. doi: 10.3389/fendo.2022.884777 (PMC9207215; doi:10.3389/fendo.2022.884777)
Supplement: Supplementary file 11 [file Table_1.docx]

**Description of supplementary materials**

Supplementary figure 1. The heat map of the expression differences of 55 CAF-related DEGs in normal and tumor samples, based on the TCGA database.

Supplementary figure 2. The univariate Cox regression results of the 22 genes related to overall survival (OS) in patients with hepatocellular carcinoma (HCC).

Supplementary figure 3. The mutation and putative copy-number alterations analysis and correlation analysis of the 22 CAF-related genes in 366 HCC samples from TCGA.

Supplementary figure 4. Least absolute shrinkage and selection operator (LASSO) Cox regression results. (A) LASSO Cox coefficients of the 22 CAF-related genes. (B) LASSO ten-fold cross-validation (C) The correlation of the 14 CAF-related genes identified by LASSO Cox regression. (D) The multivariate Cox regression results of 14 CAF-related genes identified by LASSO Cox regression.

Supplementary figure 5. The correlation of risk score and the five clinicopathological characteristics.

Supplementary figure 6. The results of univariate and multivariate Cox regression. (A) univariate Cox regression of factors including age, gender, pathologic stage, histological grade, AFP level, and prognostic risk score. (B) multivariate Cox regression of factors including age, gender, pathologic stage, and prognostic risk score.

Supplementary figure 7. The difference in tumor microenvironment scores and tumor purity between high and low risk group.

Supplementary figure 8. The result of prediction of immunotherapy response. (A) The expression level of 43 immune checkpoints in low and high risk groups. (B) The correlation between PD-1 expression and risk score. (C) The correlation between CTLA-4 expression and risk score.

Supplementary figure 9. The heat map of GSVA enrichment between low-risk and high-risk groups.

Supplementary figure 10. Survival analysis of the TOP ten hub genes. (A-I) K-M curves of nine hub genes. (J-K) The univariate and multivariate COX regression results of TOP ten hub genes, respectively.
